# Repeatability and reproducibility of a clinical device for Brillouin microscopy to measure the biomechanics of the anterior segment of the eye: In vivo tests

**DOI:** 10.1371/journal.pone.0353667

**Published:** 2026-07-20

**Authors:** Hannah Pearl Glucksman, Andrew Shin, Lawrence Yoo, Luis Alberto Carvalho, Claire Shelley Barnes

**Affiliations:** Intelon Optics, Woburn, Massachusetts, United States of America; Singapore National Eye Centre, SINGAPORE

## Abstract

**Purpose:**

We performed a prospective study to assess the repeatability and reproducibility of the novel Brillouin Optical Scanning System (BOSS^®^) in measuring the biomechanical properties of the cornea and crystalline lens in vivo, to determine device utility in a clinical setting.

**Methods:**

Adult study participants underwent standard ophthalmic exams to confirm eligibility. At two separate visits to the clinic, monocular lens scans and corneal scans (one point and seven points, respectively) were performed three times on each of three BOSS devices, operated by different technicians. The measured Brillouin parameters per person included the lens stiffness and the stiffnesses (e.g., “Mean”, “Minimum”) across the seven corneal points. Results were combined across participants to calculate the coefficients of variation (CVs) for repeatability (within each device) and reproducibility (across devices), along with the repeatability and reproducibility limits (2.8 times the respective standard deviations).

**Results:**

Eleven of the 33 participants (ages 24–72 years; 15 females) had keratoconus (5 with prior crosslinking, 3 without crosslinking, and 3 post-keratoplasty); the other 22 participants were visually normal. There were no adverse events. The average lens stiffness was 3.33 ± 0.052 GPa (mean ± SD), with an overall repeatability CV of 1.6%, with values ranging from 3.35 ± 0.037 GPa with a CV of 1.1% to 3.30 ± 0.071 with a CV of 2.1% across the separate devices. The mean corneal stiffness was 2.83 ± 0.031 GPa, with an overall repeatability CV of 1.1%, ranging from 2.85 ± 0.015 with a CV of 0.5% to 2.81 ± 0.036 with a CV of 1.3% across devices. The reproducibility CVs were 2.1% and 1.6% for lenses and corneal Means, respectively.

**Conclusions:**

The study results demonstrated consistent stiffness values for both lenses and corneas within and between the three BOSS devices tested. The study shows that this device could be a useful new biomechanics-measuring tool for applications in ophthalmology.

## Introduction

Measuring the biomechanical characteristics of the cornea and lens is important for improving the diagnosis and treatment of some ocular diseases [1–7]. Although there have been multiple articles written about the importance of biomechanics in ophthalmology [2,4–6,8], most of the in vivo techniques to measure the biomechanical properties of the eye are limited [2,4–6,9,10]. For example, some devices rely on air puffs to measure corneal biomechanics; however, this method is sensitive to intraocular pressure [8] and only indirectly quantifies *global* corneal biomechanical parameters [2,5,11–13]. Other methods involve physical contact with ocular tissue [2,4,5,9–11,14], which poses the risk of introducing artifacts and reducing measurement reproducibility, as well as limiting patient comfort [2,4–6,9–12]. In addition, the tear film may limit corneal epithelium thickness readings with devices that use ultrasound technology [15,16]. Furthermore, there are no methods currently available that provide information regarding the biomechanical properties of the crystalline lens in vivo [2,4–6,8,10,14]. These shortcomings have discouraged clinicians from acquiring the much-needed biomechanical information, which could be integrated into personalizing treatment strategies.

Brillouin ocular microscopy [17–20] addresses these weaknesses with a contactless technique for evaluating the biomechanical properties of both the cornea and crystalline lens in vivo. Brillouin light scattering quantifies tissue elasticity (‘stiffness’) at the substructural level [7,17–31]. The technique is based on the interaction between the light directed at a tissue and the acoustic phonons within it, which are distinct units of energy inside the sample; there is no need for tissue manipulation [7,17–31].

**Fig 1:**
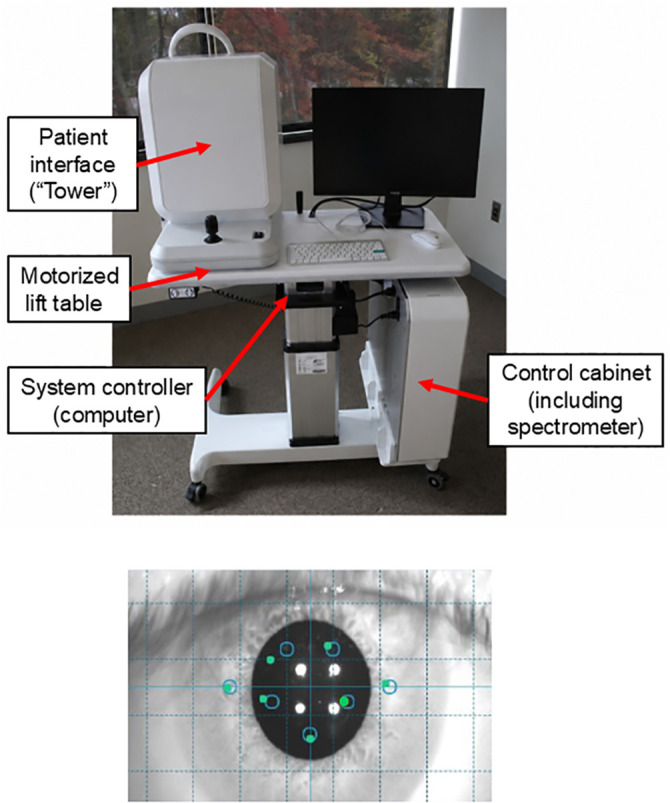
The Brillouin Optical Scanning System (BOSS). Upper: Photograph of the BOSS, as seen from the operator’s side, with the major components labeled. To stabilize head position, the patient sits with their chin on a chinrest and forehead against a forehead rest (not shown) on the far side of the Tower. The operator uses the joystick on the near side of the Tower to adjust the chinrest height for patient comfort, and uses the keyboard, mouse, and monitor to run the software for performing and reviewing scans. Lower: The 7-point corneal pattern. Once the operator initiates a scan, the BOSS automatically moves the target for scanning between the locations indicated by the open circles, progressing clockwise from the upper-left. The filled circles, which appear at the end of the scan, indicate where the scans were recorded.

We have now developed a clinical device, based on earlier research prototypes [7,17,22], to perform Brillouin microscopy within ophthalmology practices. Following our preliminary, laboratory-based research [32,33], experiments were conducted to document the repeatability and reproducibility of in vivo measurements of human corneas and lenses using the new Brillouin Optical Scanning System (BOSS^®^, Intelon Optics, Woburn, MA; Fig 1, upper). Consistency in measurements on repeated trials, across different eyes, devices, and operators, is critical in validating the reliability of any biomechanical assessment tool, to track an individual patient’s ocular health and to make comparisons against normative data [29–41]. In this study, the BOSS device showed uniform measurements in the series of repeated clinical scans, displaying repeatable and reproducible results that were consistent with the literature.

## Methods

### Brillouin microscopy overview

Brillouin microscopy has similarities to acoustic microscopy or ultrasound imaging, as the underlying principle in each method relies on acoustic propagation speeds [21,23,24]. The technologies differ in that Brillouin microscopy does not require ultrasound transducers and offers much-higher spatial resolution than does a direct measurement of acoustic wave phenomena [17,21–25]. In Brillouin scattering, laser excitation of a small volume of anterior segment tissue is produced by a scanning confocal optical system (located in the “Interface Tower”, Fig 1, upper). A fraction of the focused laser light scatters from the acoustic waves (phonons) that are present in all solid materials [21,23–25]. The scattered light experiences a small Doppler shift in frequency, which is related to the frequency of the acoustic waves in the material. The Brillouin scattered light is re-imaged by the scanning optical system and relayed to a spectrometer with a virtually imaged phase array (VIPA). The VIPA produces a series of virtual images that are sensitively frequency-dispersed [17,21,22,24,25]. The software then generates axial profiles of the cornea or lens from the single-point scans, as well as two-dimensional maps of corneas after a multi-point corneal scan (Supplementary Fig 1, SF1). The BOSS also employs digital-camera eye registration during scans, to register the elasticity maps with respect to the pupil center, to indicate, after scanning, the locations that were scanned (filled circles in Fig 1, lower). Brillouin microscopy [22] and the BOSS device itself are described further in papers by Chang et al., [42], Jeong et al., [43], and in prior ARVO abstracts [32,33,44].

### Human testing methods

The clinical study protocol was approved by Salus IRB (Protocol P22-01), and the project was listed on clinicaltrials.gov. Signed informed consent was obtained from all potential study participants, after an explanation of the nature and possible consequences of the study, before any study-specific evaluations were performed. All testing was conducted at the Vold Vision Ophthalmology clinic in Fayetteville, AR.

The planned study enrollment was 30 adults (≥18 years old), to achieve the predetermined sample size of 21 eyes for analysis of variance (ANOVA) calculations. The goal was the scanning of one eye per participant across a range of softer to harder lenses and corneas, to characterize the dynamic ranges of the BOSS as a measurement tool. Accordingly, the minimum numbers of eyes were set at: 5 non-diseased eyes of participants younger than 55, and 5 of those 55 years old or older; 3 eyes that had undergone a corneal transplant for keratoconus (KC); 3 eyes with KC without prior corneal crosslinking (CXL); and 3 that had undergone CXL for KC. The study was not powered to make comparisons between these groups. The exclusion criteria were: i) no light perception, or low vision precluding stable fixation; ii) corneal opacities or cortical cataracts visible in a non-dilated eye; and iii) systemic disease or disorder that could prohibit image acquisition (e.g., Parkinson’s disease). If both eyes of a patient qualified for enrollment, the patient was included in the group that required more eyes to complete the targeted group minimum. For participants with two non-diseased eyes, the study eye was selected based on a randomization table.

Participants were scheduled for two visits, less than 30 days apart. At the first visit, clinical data were collected to establish whether both eyes qualified for inclusion; this included documenting any ocular pathology. The binocular evaluations included intraocular pressure using Goldmann applanation tonometry [45,46], slit lamp examination, and dilated fundus exam. Once the study eye was determined, tests on that eye comprised: i) manifest refraction; ii) best corrected visual acuity, using early treatment diabetic retinopathy study (ETDRS) charts [47,48]; iii) measurement of central corneal thickness, using an ultrasonic pachymeter [45]; iv) anterior segment optical coherence tomography [40,49]; and v) Pentacam scans [50,51].

Participants underwent three BOSS lens measurements on each of three different devices operated by different trained operators, for a total of nine measurements per participant (three replicates × three devices/operators). A corresponding set of nine cornea scans, with an automated 7-point pattern (Fig 1, lower, open circles), was performed on the study eye at each participant’s second visit. Corneal scans were performed without dilation. For all scans, participants were instructed to maintain fixation on the central green light-emitting diode and ‘ignore’ the 780-nm infrared laser light as it moved around the field of view during scans. Participants were told that they could blink naturally, but to try not to do so excessively. When this study was performed, the 7-point corneal scan took approximately 3.5 minutes, and the lens scan took 30 s. Updates about progress were provided throughout the scans to encourage attention to the task.

All scanning was performed by ophthalmic technicians trained in the operation of the device, including becoming familiar with the expected appearance of lens and corneal profiles. It was at the discretion of the operator to repeat up to two more lens scans, or two more complete or partial corneal scans if the quality of a given scan was deemed inadequate, e.g., due to excessive blinking or movement.

During device development, the BOSS was tested against applicable standards to ensure the safety of the device for clinical use, following the standards described in ISO (1994) [52] and IEC (2020) [53]. In addition, the system was evaluated according to ANSI (2014) [54], due to the laser source. The Maximum Permissible Exposure for 780-nm laser energy was used as a safety reference, to ensure that controls were in place so that the energy exposure was significantly below that level during all exams.

### Statistics

Following corneal scanning with the 7-point pattern, the (up to) 7 separate stiffness values recorded, in gigapascals (GPa), were combined by BOSS to generate values for these Brillion modulus (BM) parameters for each scan: “Central BM” (the value measured closest to the center of the pupil); “Mean BM” (average across all recorded locations); “Min BM” (the lowest BM value measured); and “Max BM” (the highest BM value measured). Lens scans generated a single value, the “Lens BM.”

ANOVAs were used to determine repeatability and reproducibility, as well as the variance associated with the combination of operators and devices. The sample size (at least 21 individuals with complete data) was calculated for detecting significant variations of measurements due to the operator/device combinations (see Supplementary Table 1 (ST1)). To achieve the enrollment goal, recruitment of 30 participants was planned.

Descriptive statistics such as mean, standard deviation (SD), median, minimum, and maximum are provided for continuous variables; counts and percentages are provided for the categorical data. Modeling was performed with restricted maximum likelihood methods, as implemented in SAS version 9.4 (SAS Institute Inc., Cary, NC) or R version 4.0 (R Foundation for Statistical Computing, Vienna, Austria). Additional analyses were performed in Microsoft Excel, version 365 (Redmond, WA).

Repeatability, across scans per participant per device, and reproducibility, across operators/devices, were calculated for each of the 5 BM values listed above. We focused on the dimensionless coefficients of variation (CVs = 100 × SD/average) for the multiple output parameters, as that provides a percentage measure of the dispersal of results around the average, even with different means [55]. We also calculated the repeatability and reproducibility limits, as follows. The repeatability SD is the square root of the estimated variance due to measurement error. The repeatability limit is 2.8 times the repeatability SD and represents the measurements below which the difference between results of replicate tests (i.e., with the same operator on the same device) are expected to occur approximately 95% of the time [56]. The reproducibility SD is the square root of the sum of the variances due to device/operator configurations, the interaction between participant and device/operator configuration, and measurement error. The reproducibility limit is 2.8 times the reproducibility SD. The repeatability and reproducibility limits are measured in gigapascals (GPa), as are the original BOSS measurements, and are representative of measurement precision. No imputation of data was performed for missing BM values.

## Results

The distribution of participants/group was: 11 young normal, 11 older normal, 3 eyes post-transplant, 3 eyes with KC without CXL, and 5 eyes with KC post-CXL. The demographic details for the study participants are presented in Table 1. Among the clinical measures, there were no abnormal findings on slit-lamp examinations, and only one participant with an abnormal dilated fundus exam (posterior vitreous detachments in both eyes). The vertical cup-to-disc ratio was 0.4 ± 0.15 (mean ± SD) for both the study and fellow eyes. Additional clinical details are provided in Supplementary Tables 2 (ST2) and 3 (ST3). No adverse effects occurred during the clinical study.

**Table 1:** Study participant demographics (N = 33).

Parameter	Statistic	Values
Age (years)	Mean (SD*)	45.1 (13.4)
	Range	24–72
Sex, n (%)	Female	15 (45.5)
	Male	18 (54.5)
Race, n (%)	Black or African American	3 (9.1)
	White	25 (75.8)
	Unknown	5 (15.2)
Study Eye, n (%)	Right	14 (42.4)
	Left	19 (57.6)
Mean IOP^†^, Study Eye (mm Hg)	Mean (SD)	13.9 (3.67)
	Range	6–21
Mean CCT^‡^, Study Eye (µm)	Mean (SD)	520.5 (61.02)
	Range	323–642

* SD= standard deviation; † IOP= intraocular pressure; ‡ CCT= central corneal thickness

**Fig 2:**
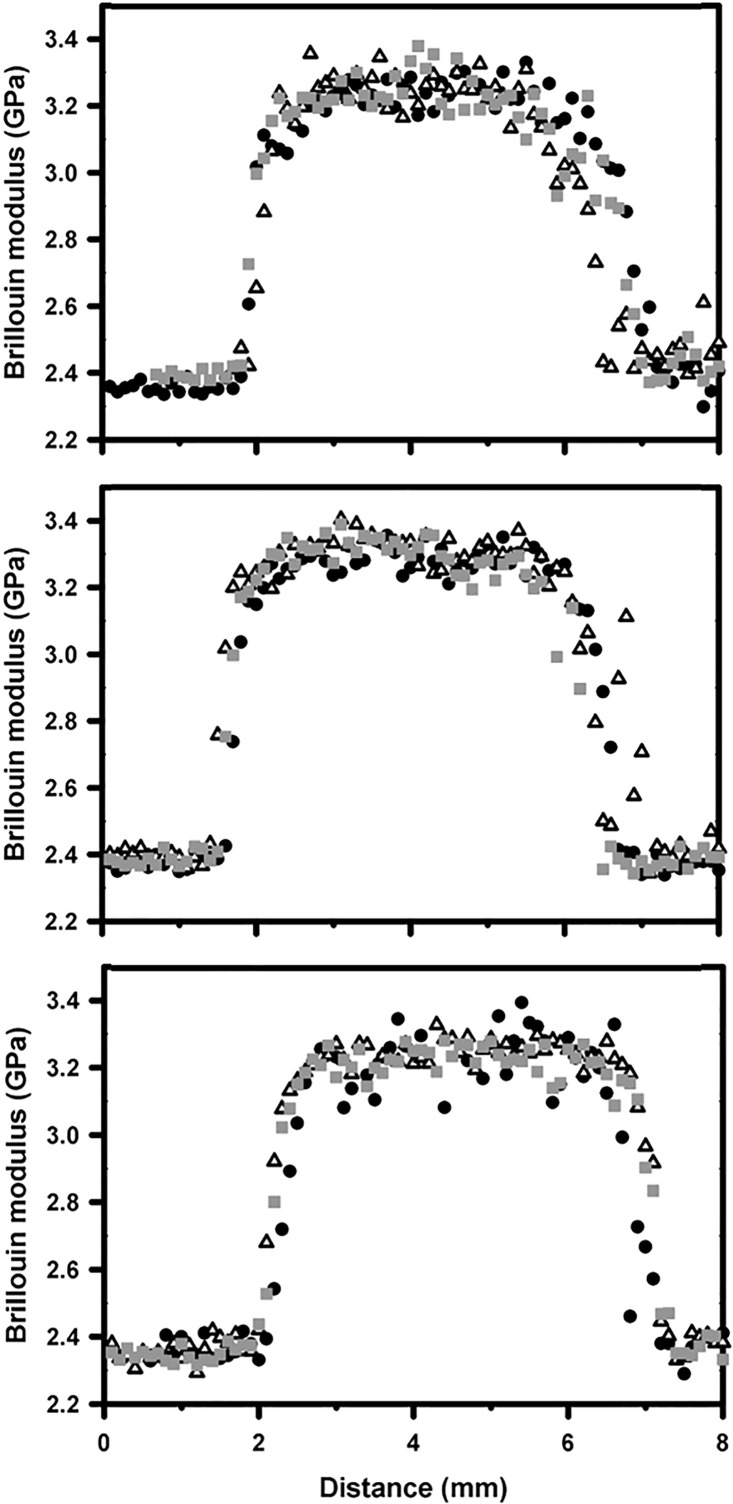
Examples of human lens stiffness profiles recorded with the Brillouin Optical Scanning System (BOSS). Scanning proceeds from the aqueous humor, on the left, through the lens to the vitreous humor on the far right. Each panel shows three scans, represented by different symbols, recorded from the left eye of a 57-year-old male. Each panel shows the results recorded on a different device, each run by a different operator. To account for slight differences in the starting depth of the scans, the profiles were shifted slightly into alignment along the x-axis; no changes were made to the y-axis values.

**Fig 3:**
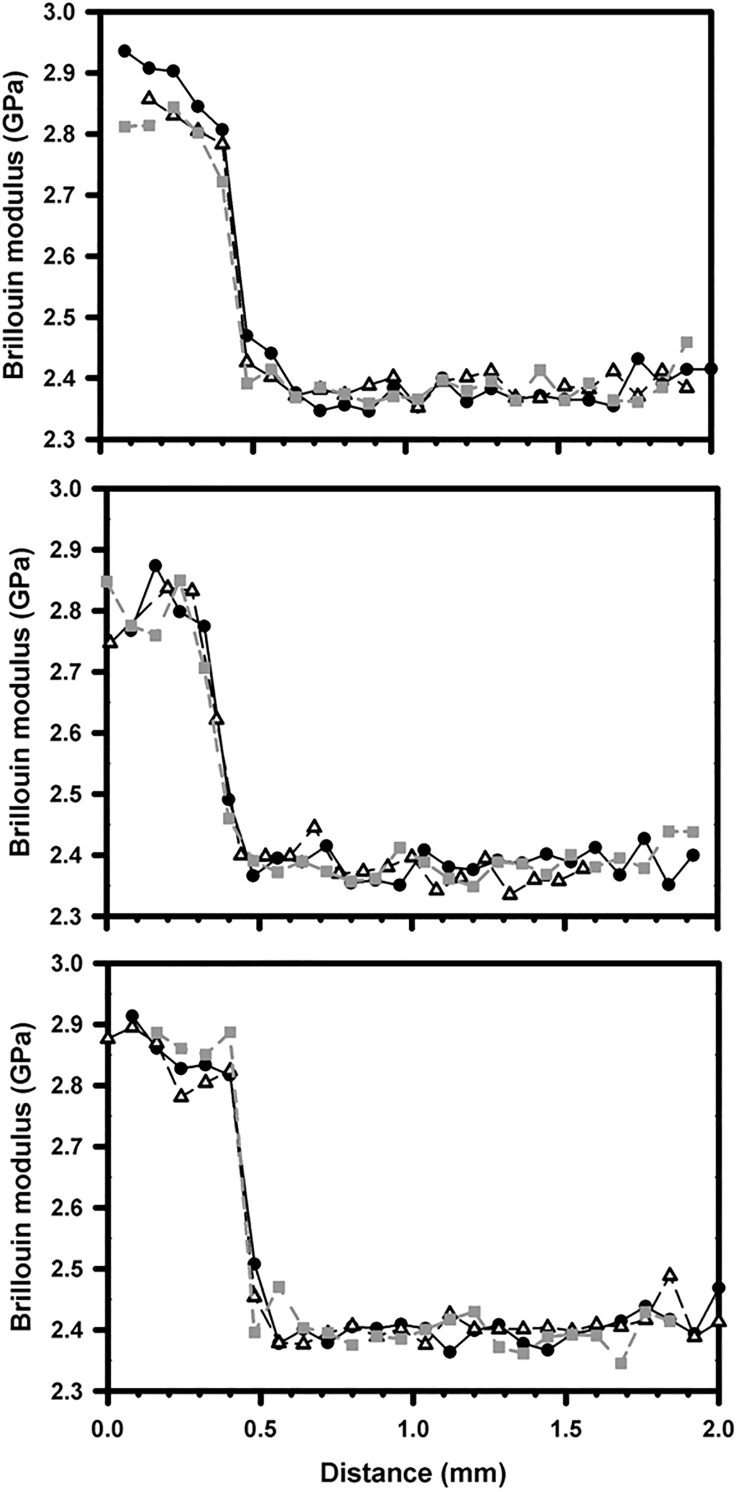
Examples of human cornea stiffness profiles recorded with the Brillouin Optical Scanning System (BOSS). Scanning proceeds from the cornea on the left to the aqueous humor on the right. Each panel shows three replicate scans, represented by different symbols, from the left eye of a 40-year-old female. The three panels show the results recorded for target location 6 (x= −1.8 mm, y= −0.6 mm, from the center of the pupil) on each of the three different devices, each run by a different operator. To account for slight differences in the starting distance of the scans from the corneal surface, the profiles were shifted slightly into alignment along the x-axis; no changes were made to the y-axis values.

Figs 2 and 3 show representative scans through a lens and through a single location on the cornea, respectively. Each panel shows three replicate scans on a single device, with the three panels per figure showing the scans from the three different BOSS devices. In each case, the variable of interest is the elasticity, in GPa, i.e., the height of the plateau through the lens or cornea, which was similar within and between panels.

In the lens study, analyzable scans were obtained from 31 of the 33 enrolled participants. The other 2 were excluded because 1 person had intraocular lenses, so could only undergo cornea scans, and 1 had nystagmus severe enough to preclude reliable scanning of the lens or cornea when attempted on the first device. With each lens scan lasting approximately 30 s, the total imaging time per participant at the first research visit was less than 30 minutes, with frequent rest periods between scans to mitigate tiredness and lapses in attention. Corneal scans were obtained from 32 participants, using the preset 7-point pattern, which was chosen to cover the cornea but not be too tiring. Of the maximum possible 288 cornea scans, some were lost, primarily due to patient fatigue and/or scheduling conflicts with the long cornea protocol at the second visit.

### Lenses

Repeatability and reproducibility of the crystalline lens measurements were characterized for each BOSS device as well as overall. Overall, the lens measurements showed an average stiffness of 3.33 ± 0.052 (mean ± SD) GPa, with average values ranging from 3.29 ± 0.071 to 3.35 ± 0.037 GPa for the individual devices (Table 2). The average CV was 1.6%, ranging from 1.1% to 2.1% across devices, and the reproducibility CV was 2.1%. The repeatability limits ranged from 0.10 to 0.20 GPa across devices, and the reproducibility limit was 0.20 GPa.

**Table 2:** Repeatability and Reproducibility of the Brillouin Moduli (GPa) of the Crystalline Lens (N = 33).

Statistic	Unit #1	Unit #2	Unit #3	Overall
Number of Eyes	30	31	31	31
Number of Scans Included in Analysis	89	92	90	271
Average	3.294	3.349	3.333	3.326
Standard Error	0.010	0.007	0.007	0.005
Repeatability SD*	0.071	0.037	0.041	0.052
Repeatability CV (%) ^**†**^	2.1	1.1	1.2	1.6
Repeatability Limit	0.198	0.104	0.116	0.145
DevOP ^‡^ SD				0.027
Reproducibility SD				0.07
Reproducibility CV (%)				2.1
Reproducibility Limit				0.195

* SD= standard deviation; † CV=coefficient of variation; ‡ DevOP= Device/Operator

### Corneas

A total of 259 cornea scans from a total of 32 patients were used for the analysis of the 4 cornea parameters on each device, as well as overall. The average value of Mean BM from cornea scans was 2.83 ± 0.031 GPa, with values from 2.81 ± 0.036 to 2.85 ± 0.015 GPa for the 3 devices (Table 3). The overall CV for Mean BM was 1.1%, with values ranging from 0.5% to 1.4% for the individual devices. The reproducibility CV was 1.6%. Across participants, the average minimal Brillouin stiffness (Min BM, Supplementary Table 4 (ST4)) and average maximal Brillouin stiffness (Max BM, Supplementary Table 5 (ST5)) were 2.78 ± 0.003 GPa and 2.90 ± 0.004 GPa, respectively. That for Central BM was 2.84 ± 0.004 GPa (Supplementary Table 6 (ST6)). The across-device repeatability CV values for the Min, Max, and Central parameters ranged from 1.4% to 2.3%, and the reproducibility CVs from 1.9% to 2.5%. As shown in Table 3 and Supplementary Tables ST4 to ST6, the repeatability limits ranged from 0.04 to 0.23 GPa, across parameters and devices, and the reproducibility limits ranged from 0.13 to 0.20 GPa.

**Table 3:** Repeatability and Reproducibility of the “Mean” Brillouin Moduli (GPa) for the 7-Point Cornea Pattern (N = 33).

Statistic	Unit #1	Unit #2	Unit #3	Overall
Number of Eyes	29	29	31	32
Number of Scans Included in Analysis	85	84	90	259
Average	2.831	2.811	2.854	2.833
Standard Error	0.006	0.005	0.003	0.003
Repeatability SD*	0.039	0.036	0.015	0.031
Repeatability CV (%) ^**†**^	1.4	1.3	0.5	1.1
Repeatability Limit	0.108	0.101	0.041	0.088
DevOP SD ^‡^				0.02
Reproducibility SD				0.046
Reproducibility CV (%)				1.6
Reproducibility Limit				0.129

* SD= standard deviation; † CV=coefficient of variation; ‡ DevOP= Device/Operator

## Discussion

The lens and corneal profiles (Figs 2 and 3) in this study showed similar intra- and inter-individual shapes across individuals. The profiles also closely resemble human Brillouin scans in the literature as recorded on the existing research devices [57,58], demonstrating the functionality of this novel Food and Drug Administration (FDA)-cleared Brillouin system. The ranges of Brillouin moduli values recorded in this trial are also consistent with earlier research studies [57,58]. These results support the accuracy conclusions reached in our bench tests [32,33]. Therefore, the next task was to determine the repeatability and reproducibility of scans performed on the device.

CV values in the literature vary substantially across test conditions, as do the values that are considered “good.” In device research in ophthalmology, multiple reports of CVs can be found, ranging from ≤1% (central corneal thickness measurements [59–61]) to ≥25% (optical coherence tomography angiography [62]), with many CVs around 5% to 7% [63–66]. Even repeatability CVs ≥ 10% have been described as “good” [35,62,66,67]. In our porcine eye repeatability results [33], the repeatability CVs were ≤1.1% and ≤1.8% for lenses and corneas, respectively, from pigs of different ages. Based on the above reports [35,59–69] and our porcine experiments [32,33,43], and allowing for the likely variability in human scans (e.g., due to eye movements), initial predictions of 20% CVs were made, although far lower values were expected. The predicted performance level was met by a large margin, not only across devices for all 4 cornea BM parameters and the Lens BM but also within each device (0.5% – 2.9%) for each parameter.

Likewise, the CVs for reproducibility across the 3 devices can be considered quite low (≤ 2.5%), relative to the literature, despite the various meanings of “reproducibility” [35,36,39,60–73]. Specifically, most reports use the term to refer to repeated testing on a single device with different operators and/or on different days. Conversely, there have been few descriptions of reproducibility measured on different examples of the same instrument, as here with our 3 BOSS devices. However, the reproducibility CVs of 1.2% to 7.9% from the measurement of dynamic corneal response parameters on 3 Corvis ST devices [70] are comparable to, or a bit higher than our inter-device reproducibility.

The repeatability and reproducibility limits are defined as 2.8 times the repeatability and reproducibility SDs [52]. Due to the different measurement units reported for such limits, it is not possible to make a direct comparison with those in previous studies, but the present values are small (around 0.1 to 0.2 GPa). Altogether, the repeatability and reproducibility seen with the BOSS device are well-aligned with accepted values in earlier ophthalmic-device studies.

A small subgroup analysis was performed on the cornea data, to determine whether the repeatability results were affected by the types of individuals tested (further explained in Supplementary Tables ST7 and ST8). The 1-way ANOVA for Mean BMs showed a P-value of 0.04, with the only significant pairwise difference being higher CVs in the transplant group than in the Older control group (Tukey-Kramer test). A separate 1-way ANOVA showed no significant differences between groups in Max BM CoVs (P = 0.24; Supplementary Fig. SF2). Together, these results (with small and disparate numbers of people per group) suggest that scan variability may be slightly higher for the transplant group but generally do not differ much between groups. This suggestion is consistent with similar repeatability measurements in an earlier study involving control subjects and patients with KC [74].

Only a single device was used for our preliminary ex vivo experiments, so reproducibility data are not available for comparison between the ex vivo and in vivo experiments. However, it is noteworthy that the CVs for human BM repeatability measurements were not much higher than those from the experiments with the mounted (i.e., stationary, unblinking) porcine eyeballs. Although the present experiments were not designed to compare results between species, it is interesting that our Mean corneal BM values were higher than our ex vivo corneal values (~2.83 versus ~2.75 GPa, respectively). That result is consistent with prior measurements obtained with different methods [75,76]. This provides additional support for the accuracy of BOSS scans. It is less apparent from the literature whether porcine- or human-lens BOSS values should be higher, and the striking difference in shape (i.e., a peaked shape for porcine lenses [33]) further confounds the comparison. However, Figs 4 and 5 in Schumacher et al. [77] showed higher GHz values for Brillouin scanning of a porcine lens in situ versus the in vivo lens of a 54-year-old, and comparisons of Fig 4 in Scarcelli et al., 2011 [24], and Fig 4 in Scarcelli et al., 2012 [22], presumably recorded on similar prototype devices, show higher BM values for pig lenses than human ones. These results are consistent with our finding of higher peak BM values in porcine lens than human ones.

Serial testing, across different devices and operators, indicated that the new device can reliably deliver precise measurements of corneal and lens biomechanics, thus supporting its use as an imaging and measurement tool [34,36,71,72]. The absence of adverse effects during this clinical trial attests to the safety of the device, making it a viable tool for ophthalmologists seeking accurate, non-invasive assessments of ocular tissue stiffness [6,34,36,40,72,73]. Other researchers have already demonstrated clinical utility for the device through a study on Small Incision Lenticule Extraction (SMILE) alone versus SMILE with collagen cross-linking (SMILE CXL) on corneas [78].

However, there are some key limitations in this study. First, only intra-visit comparisons were made. In future studies, inter-visit data must be collected to establish the consistency of results across time [64,68], and, thereby, to determine what are significant changes in corneal biomechanics, e.g., to document when true progression occurs in KC. Second, there was a small population in this study; however, a wide range of ages (24–72 years) and ocular characteristics (Table 1, and Supplementary Tables ST2 and ST3) were represented in the study. Our findings expand upon recent studies with BOSS devices in other countries, which have shown good repeatability results for lens scans [79] and 10-point cornea patterns [74], and we now add reproducibility results. Third, only a small proportion of the individuals tested in the study had KC, treated or untreated, so no analysis of the BM values between the groups wasperformed. Due to the potential applications of the BOSS in detecting and/or following KC, it is important to test a larger population of patients with KC to determine the sensitivity of BOSS in distinguishing between a control group and a group of patients with KC, although a preliminary study showed promise [74], consistent with research-prototype studies [7].

A final limitation in this study was the complex protocol, with relatively long durations of the scans, particularly the corneal scans. At the time this study was carried out, each scan through a single location (i.e., through the lens or a single corneal location) lasted approximately 30 s. However, with the expectation that standard clinical testing would only require only one or two scans per eye, and particularly with the recent technical improvements to shorten the corneal scan durations by half, this problem should be minimized. In the meantime, clinical trials have begun at several sites, to prospectively collect BOSS cornea and lens data from visually normal participants and patients with ocular disorders. These studies will supplement the normative data from our study and provide information regarding the sensitivity of the BOSS across disorders [42,43,80–83].

These findings, collectively, suggest that the BOSS device achieved satisfactory results for biomechanical measurements in ex vivo conditions [32,33,44] as well as showing substantial promise for in vivo use. The ability to provide consistent and dependable data across different settings and operators is critical for the adoption of this technology in clinical practice.

## Supporting information

S1 Table Analysis of variance for study design. (DOCX)

S2 Table Manifest refraction findings for the study eyes at Visit 1. (DOCX)

S3 Table Monocular (study eye) best-corrected visual acuities at Visit 1. (DOCX)

S4 Table Repeatability and reproducibility of the “Central” Brillouin Moduli for the 7-point cornea pattern. (DOCX)

S5 Table Repeatability and reproducibility of the “Minimum” Brillouin Moduli for the 7-point cornea pattern. (DOCX)

S6 Table Repeatability and reproducibility of the “Maximum” Brillouin Moduli for the 7-point cornea pattern. (DOCX)

S7 Table Subgroup analyses of “Mean BM” (GPa) repeatability %CVs for the 7-point cornea pattern. (DOCX)

S8 Table Subgroup analyses of “Max BM” (GPa) repeatability %CVs for the 7-point cornea pattern. (DOCX)

S1 Fig Example scan-review screen on BOSS^®^. (DOCX)

S2 Fig Percent coefficient of variation of Max BMs. (DOCX)
