# Supplementary material for: Repeatability and reproducibility of a clinical device for Brillouin microscopy to measure the biomechanics of the anterior segment of the eye: In vivo tests
Source: PLoS One. 2026 Jul 20;21(7):e0353667. doi: 10.1371/journal.pone.0353667 (PMC13384280; doi:10.1371/journal.pone.0353667)
Supplement: S1 Table — (DOCX) [file pone.0353667.s001.docx]

### Sample size calculation:

With 21 eyes, if there are no missing observations, the analysis of variance (ANOVA) degrees of freedom for this study are summarized as follows.

### **Supplementary Table 1:** Analysis of variance (ANOVA) for study design

| **Source** | **Degrees of Freedom (DoF)** |
| --- | --- |
| Eye | 20 |
| Device/Operator | 2 |
| Eye × Device/Operator | 40 |
| Error = Rep(Eye, Device/Operator) | 188 |

With at least 21 subjects with complete data, we would have 188 DoF for the repeatability estimate, to produce a confidence interval slightly shorter than 91% to 112% of the estimate.

To ensure complete data from 21 eyes, the study set out to enroll 30 subjects (one eye per subject).
