# Supplementary material for: Repeatability and reproducibility of a clinical device for Brillouin microscopy to measure the biomechanics of the anterior segment of the eye: In vivo tests
Source: PLoS One. 2026 Jul 20;21(7):e0353667. doi: 10.1371/journal.pone.0353667 (PMC13384280; doi:10.1371/journal.pone.0353667)
Supplement: S2 Table — (DOCX) [file pone.0353667.s002.docx]

**Supplementary Table 2:** Manifest refraction (MR) findings for the study eyes at Visit 1 (N=33)

| **Parameter** | **Statistic** | **Study eye** |
| --- | --- | --- |
| MR Sphere | Mean (SD*) | −2.37 (3.421) |
|  | Median | −1.00 |
|  | Min, Max | −12.25, 1.00 |
|  |  |  |
| MR Cylinder | Mean (SD) | 1.29 (1.711) |
|  | Median | 0.75 |
|  | Min, Max | 0.00, 8.00 |
|  |  |  |
| MR Axis | Mean (SD) | 80.91 (66.495) |
|  | Median | 90.00 |
|  | Min, Max | 0.00, 180.00 |

* SD= standard deviation
