# Supplementary material for: Repeatability and reproducibility of a clinical device for Brillouin microscopy to measure the biomechanics of the anterior segment of the eye: In vivo tests
Source: PLoS One. 2026 Jul 20;21(7):e0353667. doi: 10.1371/journal.pone.0353667 (PMC13384280; doi:10.1371/journal.pone.0353667)
Supplement: S3 Table — (DOCX) [file pone.0353667.s003.docx]

**Supplementary Table 3:** Monocular (study eye) best-corrected visual acuities (BCVAs) at Visit 1 (N=33)

| **BCVA*** | **Study Eye; n (%)** |
| --- | --- |
| **20/12.5 or better** | 0 (0) |
| **20/15 or better** | 3 (9.1) |
| **20/20 or better** | 23 (69.7) |
| **20/25 or better** | 25 (75.8) |
| **20/32 or better** | 29 (87.9) |
| **20/40 or better** | 31 (93.9) |
| **20/50 or better** | 32 (97.0) |
| **20/63 or better** | 33 (100) |
| **20/80 or better** | 33 (100) |
