# Supplementary material for: Repeatability and reproducibility of a clinical device for Brillouin microscopy to measure the biomechanics of the anterior segment of the eye: In vivo tests
Source: PLoS One. 2026 Jul 20;21(7):e0353667. doi: 10.1371/journal.pone.0353667 (PMC13384280; doi:10.1371/journal.pone.0353667)
Supplement: S4 Table — (DOCX) [file pone.0353667.s004.docx]

**Supplementary Table 4:** Repeatability and reproducibility of the “Central” Brillouin Moduli (GPa) for the 7-point cornea pattern (N=33)

| **Statistic** | **Unit #1** | **Unit #2** | **Unit #3** | **Overall** |
| --- | --- | --- | --- | --- |
| Number of Eyes | 29 | 29 | 31 | 32 |
| Number of Scans Used in Analysis | 85 | 84 | 90 | 259 |
| Average | 2.843 | 2.817 | 2.855 | 2.839 |
| Standard Error | 0.008 | 0.009 | 0.005 | 0.004 |
| Repeatability SD* | 0.072 | 0.081 | 0.042 | 0.067 |
| Repeatability %CV **^†^** | 2.5 | 2.9 | 1.5 | 2.3 |
| Repeatability Limit | 0.202 | 0.226 | 0.116 | 0.186 |
| DevOP SD ^‡^ |  | | | 0.019 |
| Reproducibility SD |  |  |  | 0.070 |
| Reproducibility %CV |  |  |  | 2.5 |
| Reproducibility Limit |  |  |  | 0.197 |

* SD= standard deviation; **^†^** CV=coefficient of variation; ^‡^ DevOP= Device/Operator
