# Supplementary material for: Repeatability and reproducibility of a clinical device for Brillouin microscopy to measure the biomechanics of the anterior segment of the eye: In vivo tests
Source: PLoS One. 2026 Jul 20;21(7):e0353667. doi: 10.1371/journal.pone.0353667 (PMC13384280; doi:10.1371/journal.pone.0353667)
Supplement: S5 Table — (DOCX) [file pone.0353667.s005.docx]

**Supplementary Table 5.** Repeatability and reproducibility of the “Minimum” Brillouin Moduli (GPa) for the 7-point cornea pattern (N=33)

| **Statistic** | **Unit #1** | **Unit #2** | **Unit #3** | **Overall** |
| --- | --- | --- | --- | --- |
| Number of Eyes | 29 | 29 | 31 | 32 |
| Number of Scans Included in Analysis | 85 | 84 | 90 | 259 |
| Average | 2.777 | 2.738 | 2.811 | 2.776 |
| Standard Error | 0.007 | 0.005 | 0.004 | 0.003 |
| Repeatability SD* | 0.048 | 0.043 | 0.023 | 0.039 |
| Repeatability CV (%) **^†^** | 1.7 | 1.6 | 0.8 | 1.4 |
| Repeatability Limit | 0.134 | 0.121 | 0.065 | 0.110 |
| DevOP SD ^‡^ |  | | | 0.036 |
| Reproducibility SD |  |  |  | 0.060 |
| Reproducibility CV (%) |  |  |  | 2.2 |
| Reproducibility Limit |  |  |  | 0.168 |

* SD= standard deviation; **^†^** CV=coefficient of variation; ^‡^ DevOP= Device/Operator
