# Supplementary material for: Repeatability and reproducibility of a clinical device for Brillouin microscopy to measure the biomechanics of the anterior segment of the eye: In vivo tests
Source: PLoS One. 2026 Jul 20;21(7):e0353667. doi: 10.1371/journal.pone.0353667 (PMC13384280; doi:10.1371/journal.pone.0353667)
Supplement: S6 Table — (DOCX) [file pone.0353667.s006.docx]

**Supplementary Table 6.** Repeatability and reproducibility of the “Maximum” Brillouin Moduli (GPa) for the 7-point cornea pattern (N=33)

| **Statistic** | **Unit #1** | **Unit #2** | **Unit #3** | **Overall** |
| --- | --- | --- | --- | --- |
| Number of Eyes | 29 | 29 | 31 | 32 |
| Number of Scans Included in Analysis | 85 | 84 | 90 | 259 |
| Average | 2.896 | 2.891 | 2.901 | 2.896 |
| Standard Error | 0.007 | 0.007 | 0.004 | 0.004 |
| Repeatability SD* | 0.050 | 0.058 | 0.036 | 0.049 |
| Repeatability CV **^†^** | 1.7 | 2.0 | 1.2 | 1.7 |
| Repeatability Limit | 0.141 | 0.163 | 0.101 | 0.137 |
| DevOP SD ^‡^ |  | | | 0.000 |
| Reproducibility SD |  |  |  | 0.055 |
| Reproducibility CV |  |  |  | 1.9 |
| Reproducibility Limit |  |  |  | 0.153 |

* SD= standard deviation; **^†^** CV=coefficient of variation; ^‡^ DevOP= Device/Operator
