# Supplementary material for: Repeatability and reproducibility of a clinical device for Brillouin microscopy to measure the biomechanics of the anterior segment of the eye: In vivo tests
Source: PLoS One. 2026 Jul 20;21(7):e0353667. doi: 10.1371/journal.pone.0353667 (PMC13384280; doi:10.1371/journal.pone.0353667)
Supplement: S7 Table — (DOCX) [file pone.0353667.s007.docx]

**Supplementary Table 7:** Subgroup analyses of “Mean BM” (GPa) repeatability %CVs for the 7-point cornea pattern (N=33 subjects)

| **Group** | **Unit #1 *** | **Unit #2** | **Unit #3** | **Overall** |
| --- | --- | --- | --- | --- |
| KC without CXL | 1.21% | 0.99% | 0.95% | 1.56% |
| KC after CXL | 1.56% | 1.45% | 0.83% | 1.79% |
| KC after transplant | 2.04% | 2.39% | 0.87% | 1.85% |
| Young Controls | 1.54% | 1.44% | 0.81% | 1.40% |
| Older Controls | 1.65% | 1.12% | 0.74% | 1.60% |

***** Analysis: We calculated the average and corresponding standard deviation, and then CoV across all scans per device per Group (e.g., for the Mean BM values on Unit 1 for all older controls). This was derived separately for each group across each device and then for all devices (“Overall”). The number of scans averaged together per group per device ranged from 5 (post-CXL on device 1) to 33 (older controls on device 1) because of the varied numbers of participants per group (see Text).

Abbreviations: CV= coefficient of variation; Mean BM= average Brillouin modulus; GPa= gigapascals; n= number of scans in the analysis; KC= keratoconus; CXL= corneal crosslinking
