# Supplementary material for: Repeatability and reproducibility of a clinical device for Brillouin microscopy to measure the biomechanics of the anterior segment of the eye: In vivo tests
Source: PLoS One. 2026 Jul 20;21(7):e0353667. doi: 10.1371/journal.pone.0353667 (PMC13384280; doi:10.1371/journal.pone.0353667)
Supplement: S1 Fig — (DOCX) [file pone.0353667.s009.docx]

**Supplementary Figure 1:** Example scan-review screen on BOSS^®^. The eye photograph on the left shows the locations of the 7 targets in the cornea scan (open white circles) overlaid with filled green circles that indicate where the scans actually occurred. On the right side are the results of a left-eye scan, presented in the form of a heatmap. The colors are based on the scale of gigapascals (GPa’s) on the far-right; the stiffnesses range from 2.6 for dark red up to 2.95 for blue. On the far-left of the screen is the list of all scans for this person.

**

**
