# Supplementary material for: Repeatability and reproducibility of a clinical device for Brillouin microscopy to measure the biomechanics of the anterior segment of the eye: In vivo tests
Source: PLoS One. 2026 Jul 20;21(7):e0353667. doi: 10.1371/journal.pone.0353667 (PMC13384280; doi:10.1371/journal.pone.0353667)
Supplement: S2 Fig — (DOCX) [file pone.0353667.s010.docx]

**Supplementary Figure 2:** Percent coefficient of variation of Max BMs

**
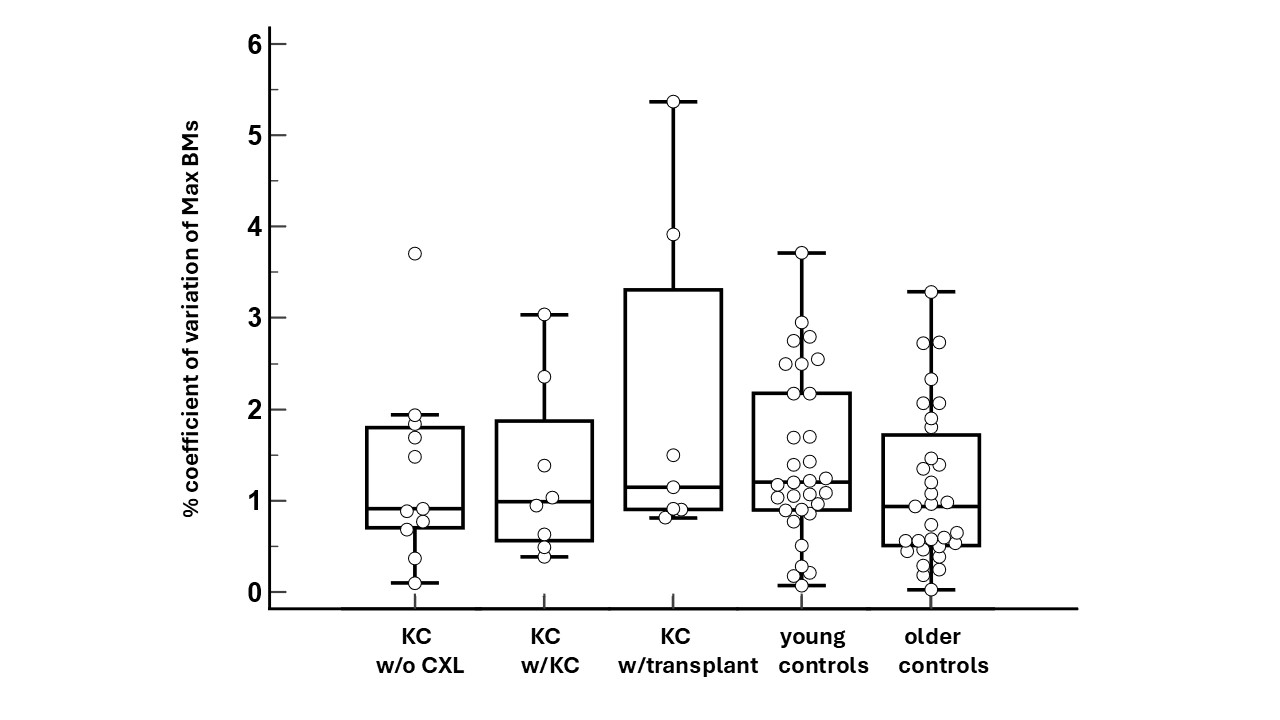
**

Supplementary Figure 2: % coefficient of variation [= (standard deviation/ average) * 100] for all cornea scans per device, grouped by participant age and ocular status.

Max BM= maximum Brillouin modulus (in gigapascals); KC= keratoconus; w/o= without; CXL= corneal crosslinking
